# Compound Defects in Halide Perovskites: A First-Principles Study of CsPbI_3_

**DOI:** 10.1021/acs.jpcc.2c06789

**Published:** 2023-01-05

**Authors:** Haibo Xue, José Manuel Vicent-Luna, Shuxia Tao, Geert Brocks

**Affiliations:** † Materials Simulation & Modelling, Department of Applied Physics, Eindhoven University of Technology, P.O. Box 513, 5600MBEindhoven, The Netherlands; ‡ Center for Computational Energy Research, Department of Applied Physics, Eindhoven University of Technology, P.O. Box 513, 5600MBEindhoven, The Netherlands; § Computational Materials Science, Faculty of Science and Technology and MESA+ Institute for Nanotechnology, University of Twente, P.O. Box 217, 7500AEEnschede, The Netherlands

## Abstract

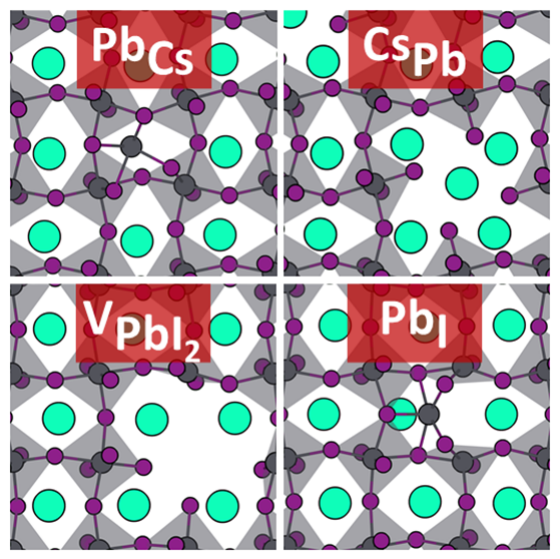

Lattice defects affect the long-term stability of halide perovskite solar cells. Whereas simple point defects, i.e., atomic interstitials and vacancies, have been studied in great detail, here we focus on compound defects that are more likely to form under crystal growth conditions, such as compound vacancies or interstitials, and antisites. We identify the most prominent defects in the archetype inorganic perovskite CsPbI_3_, through first-principles density functional theory (DFT) calculations. We find that under equilibrium conditions at room temperature, the antisite of Pb substituting Cs forms in a concentration comparable to those of the most prominent point defects, whereas the other compound defects are negligible. However, under nonequilibrium thermal and operating conditions, other complexes also become as important as the point defects. Those are the Cs substituting Pb antisite, and, to a lesser extent, the compound vacancies of PbI_2_ or CsPbI_3_ units, and the I substituting Cs antisite. These compound defects only lead to shallow or inactive charge carrier traps, which testifies to the electronic stability of the halide perovskites. Under operating conditions with a quasi-Fermi level very close to the valence band, deeper traps can develop.

## Introduction

On the basis of their outstanding efficiency (25.7% to date)^1^ and relative ease of fabrication, halide perovskite solar cells seem to be poised for large scale applications. The primary obstacle blocking their present commercialization is their relative rapid degradation under operating conditions.^2−5^ On a microscopic level, lattice defects in the perovskite materials initiate the degradation process, as they facilitate migration of ions,^6−10^ chemical reactions,^11,12^ phase transitions,^13^ and phase segregation.^14^

Because of the experimental difficulties in characterizing defect structures microscopically, much of our current understanding of lattice defects in halide perovskites stems from results obtained from electronic structure calculations based on density functional theory (DFT). Following common semiconductor practice,^15,16^ elementary defects consisting of single atomic interstitials, vacancies, or antisites have been at the center of interest.^17−21^ In a previous work,^22^ we have studied vacancy and interstitial point defects in six primary Pb- and Sn-based halide perovskites with different cations (Cs, MA, FA) and anions (I, Br, Cl), within a single computational framework.^23^ One prevalent conclusion from most of these computational studies is that in these materials the point defects with the highest concentrations under equilibrium growth conditions, only introduce shallow traps.

Defects in halide perovskites with a more complex structure have also been considered.^24−26^ Conceptually, such complex defects can be thought of as resulting from a recombination of simple atomic point defects (vacancies or interstitials) to, for instance, PbI_2_ or MAI compound vacancies in MAPbI_3_.^24−26^ Within this line of thought also antisites can be interpreted as compound defects, resulting from a recombination of an interstitial and a vacancy of different species. For instance, in CsPbI_3_, cation antisites result from a recombination of a Cs vacancy (interstitial) and a Pb interstitial (vacancy).^20^ Some compound defects have been predicted to form shallow defects only,^20,25^ whereas others have the potential to form deep traps.^24,27^

The formation energy of compound defects is typically much higher than that of simple point defects, which implies that under normal equilibrium conditions (room temperature, atmospheric pressure) the concentration of compound defects, including antisites, is negligible.^15−18^ In fact, assuming that a perovskite is formed under equilibrium conditions at room temperature, then often even the concentration of point defects is quite low.^22^ However, many crystal growth conditions are highly nonequilibrium (involving a high temperature annealing step, for instance), and defects can be formed during growth in appreciable concentrations. In molecular dynamics simulations that use a reactive force field,^10,28^ applied to halide perovskites containing an appreciable amount of point defects, one often observes recombination of the latter to compound defects. From positron annihilation lifetime spectroscopy, assisted by DFT calculations, there is evidence of charge carrier trapping at compound vacancy defects in MAPbI_3_.^26^

In this paper, we study compound defects, vacancies, interstitials, and antisites, in the archetype inorganic perovskite CsPbI_3_ by means of first-principles DFT calculations. Already this simplest of the halide perovskite compounds exhibits a wide variety of possible compound defects. Mapping out their relative likelihood of formation provides information potentially applicable to the whole class of halide perovskite compounds. Not only do we calculate the equilibrium concentrations of compound defects, but through explicitly considering the possible recombination reactions of elementary point defects, we also assess their concentrations under nonequilibrium conditions. The effect of these compound defects on the electronic properties is examined, in particular their potential to form deep traps.

## Computational Methods

### DFT Calculations

Density functional theory (DFT) calculations are performed with the Vienna *Ab-Initio* Simulation Package (VASP),^29−31^ employing the SCAN+rVV10^32^ functional for electronic calculations and geometry optimization. Our calculations use a plane wave kinetic energy cutoff of 500 eV and a Γ-point-only *k*-point mesh. The energy and force convergence criteria are set to 10^–4^ eV and 0.02 eV/Å, respectively. The SCAN functional has a superior overall performance concerning binding/formation energies over a wide range of materials and bonding configurations; for a summary, see ref (23) and references therein. In addition, as discussed in ref (23), inclusion of van der Waals interactions is important for obtaining accurate energies in lead iodide perovskites. As the SCAN functional is numerically somewhat more demanding than more traditional density functionals,^33^ we have performed additional convergence tests of defect formation energies (DFEs) with respect to increasing the kinetic energy cutoff for the plane wave basis set. The results, as shown in the Supporting Information, Figure S1, demonstrate that the DFEs calculated with the present cutoff are converged to within 0.01 eV. Spin–orbit coupling is omitted, as it has little effect on the formation energies of the most prominent defects.^23^ Spin polarization is included in all calculations.

The SCAN+rVV10 functional has a similar band gap problem as more conventional semilocal functionals, which makes it more difficult to establish the energies of defect levels with respect to the band edges.^16,34^ Hybrid functionals, fitted to reproduce the experimental band gap, may reduce this problem, and improve the description of certain classes of defect levels.^19,35,36^ Benchmarking this semiempirical approach by comparison to results obtained by beyond-DFT/Hartree–Fock techniques, such as *GW*,^37^ suffers from the fact that those results critically depend on the technical details of such calculations.^38^ More importantly in the present context, hybrid functionals can worsen the description of the thermochemistry.^16^ As the latter is a key concern in this paper, we have chosen not to use a hybrid functional. Note moreover that under thermodynamic conditions, i.e., at thermal equilibrium, the position of the Fermi level is determined by the charge neutrality condition, to be discussed below, and under that condition the defect formation energies do not depend upon the band gap, or explicitly, not on the positions of the band edges.^23,37^

As in our previous work,^22^ point defects or compound defects are created in a 2 × 2 × 2 orthorhombic supercell of CsPbI_3_, which contains 32 formula units. The lattice volume and ionic positions of the pristine supercell are fully relaxed. Within the supercell, atomic positions of defective structures are optimized. As compound defects are typically larger than simple point defects, we have performed additional tests regarding the size of the supercell, see Supporting Information, Table S1, and have concluded that the 2 × 2 × 2 supercell is sufficiently large.

### Defect Formation Energy

Under equilibrium conditions, the concentrations of lattice defects can be obtained from Boltzmann statistics
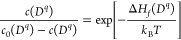
1where *D*^*q*^ indicates the type of defect, either a simple interstitial or vacancy point defect, or a compound interstitial or vacancy, or an antisite defect, with charge *q*; *c* is the defect concentration, and *c*_0_ is the density of possible sites for that particular defect (including orientational degrees of freedom if the defect is not spherically symmetric), where usually *c* ≪ *c*_0_; Δ*H*_*f*_ is the DFE, *T* is the temperature, and *k*_*B*_ is the Boltzmann constant.

Different types of defects have different charges, but if no external charges are injected, then as a whole a material has to be charge neutral
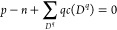
2where *p* and *n* are the intrinsic charge densities of holes and electrons of the semiconductor material. The charge neutrality condition, eq 2, fixes the intrinsic Fermi level.

The DFE is calculated from the expression^15^
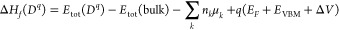
3where *E*_tot_(*D*^*q*^) and *E*_tot_(bulk) are the DFT total energies of the defective and pristine supercells, respectively, and *n*_*k*_ and *μ*_*k*_ are the number of atoms and chemical potential of atomic species *k* added to (*n*_*k*_ > 0) or removed from (*n*_*k*_ < 0) the pristine supercell in order to create the defect. We use the chemical potentials *μ*_*k*_; *k* = Cs, Pb, I, as determined for I-medium conditions in our previous work.^22^

Creating a charge *q* requires taking electrons from or adding them to a reservoir at a fixed Fermi level. The latter is calculated as *E*_*F*_ + *E*_VBM_, with 0 ≤ *E*_*F*_ ≤ *E*_*g*_, the band gap, and *E*_VBM_ the energy of the valence band maximum. As it is difficult to determine the latter from a calculation on a defective cell, one establishes *E*_VBM_ in the pristine cell, shifted by Δ*V*, which is calculated by lining up the core level on an atom in the pristine and the neutral defective cell that is far from the defect.^15,39^ As shown in ref (19), the typical supercell used in calculations and the dielectric screening in lead iodide perovskites are sufficiently large, so the electrostatic interaction between a charged defect and its periodically repeated images can be neglected, consistent with our previous work.^22,23^ In addition, we neglect vibrational contributions to the DFEs, and the effect of thermal expansion on the DFEs, as these are typically small in the present compounds.^23,40^

### Recombination Reaction

We model the recombination of point defects *A*_1_, ..., *A*_*m*_ to a compound defect *B* as a chemical reaction
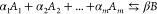
4Reaction equilibrium is defined by
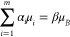
5with *μ*_*i*_ and *μ*_*B*_ the chemical potentials of species *A*_*i*_ and *B*, given by
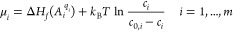
6
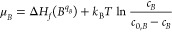
7where Δ*H*_*f*_(*D*^*q*^);  are the DFEs according to eq 3, ,  are concentrations, and ,  are the densities of possible sites (see Table S2 of the Supporting Information for details). Note that we do not assume that charge is conserved in reaction 4. The electron reservoir with Fermi energy *E*_*F*_ can supply electrons or holes, which is accounted for in the DFEs. Equations 5–7 give the law of mass action
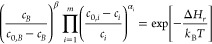
8
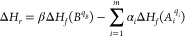
9where Δ*H*_*r*_ is the reaction energy of reaction 4.

If all (simple and compound) defects are in equilibrium with reservoirs at chemical potentials *μ*_*k*_, eq 3, then their concentrations are given by eq 1, and trivially obey the law of mass action, eq 8. Typically, however, point defects and compound defects are initially created at concentrations  and , respectively, in a crystal growth process, after which the crystals are extracted and kept at room temperature. The defects then remain, but they can recombine according to eq 4. Not only does this include the possible formation of compound interstitials or vacancies, but also the formation of antisites through the recombination of an interstitial and a vacancy.

As the recombination reaction, eq 4, conserves the total number of atoms of each species, one has
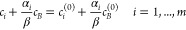
10Given the initial concentrations  and , the law of mass action, eq 8, then allows for determining the actual concentrations of the compound defect *c*_*B*_, and of the point defects *c*_*i*_.

### Charge State Transition Level

Under operating conditions, charges are injected in the material, shifting the positions of the (quasi) Fermi levels for electrons and holes. The charge state transition level (CSTL) ε(*q*/*q*′) is defined as the Fermi level position where the charge states *q* and *q*′ of the same type of defect have equal formation energy, Δ*H*_*f*_(*D*^*q*^) = Δ*H*_*f*_(*D*^*q*^′). As the DFEs have a simple linear dependence on *E*_*F*_, eq 3, this condition can be expressed as
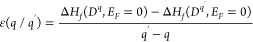
11where Δ*H*_*f*_(*D*^*q*^, *E*_*F*_ = 0) is the DFE calculated at *E*_*F*_ = 0. The CSTLs are important for the electronic properties; if these levels are deep inside the band gap, then they can trap charge carriers and act as nonradiative recombination centers.

Being based on total energies, the CSTLs calculated with SCAN+rVV10 should be fairly reliable. The positions of the band edges calculated with SCAN+rVV10 suffer from the DFT band gap error. However, we would argue that the positions of the CSTLs with respect to the band edges are correct, because the defects’ electronic states have a character similar to either the valence band or the conduction band.^41^ For a more detailed discussion, see ref (22).

## Results and Discussion

We consider different possible compound complexes, which are selected as follows. For antisite defects, we use the notation *A*_*B*_ to indicate that atom *A* substitutes atom *B* in the lattice. All six antisites are included in our selection, i.e., the cation–cation antisites Cs_Pb_ and Pb_Cs_ and the cation–anion antistes Cs_I_, I_Cs_, Pb_I_, and I_Pb_. In addition, we consider the compound antisite [2Cs]_Pb_. Of the compound interstitials and vacancies, we focus on those that correspond to formula units of the precursor materials PbI_2_ and CsI, and of the perovskite CsPbI_3_. Finally, as suggested in ref (42), we investigate the Pb_Cs_Cs_Pb_ complex, which basically is an exchange in the lattice between two neighboring Cs and Pb cations.

### Equilibrium Conditions

**Figure 1:**
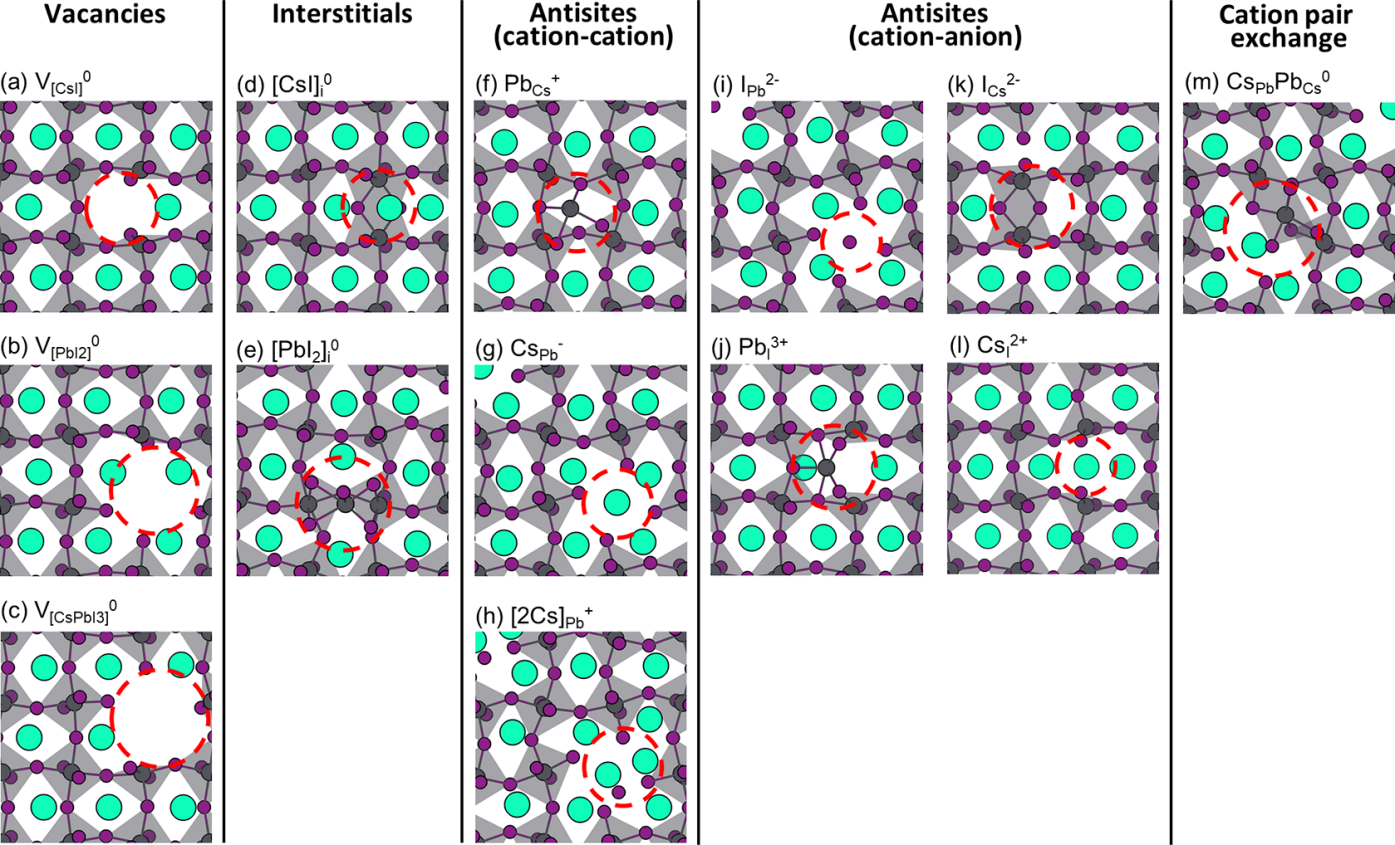
Optimized structures of compound defects in CsPbI_3_ in their most stable charge states under intrinsic conditions; (a–c) compound vacancies, (d, e) compound interstitials, (f–h) cation–cation antisites, (i–l) cation–anion antisites, and (m) cation pair exchange. Cs, Pb, and I atoms are represented by green, black, and purple circles, respectively, with PbI octahedra colored gray. The positions of the compound defects are indicated by the red circles.

Formation of compound defects in semiconductors is often driven by the attractive electrostatic interaction between defects with opposite charge states.^15,16^ Possible compound vacancies in CsPbI_3_, resulting from recombination of the point vacancies , , and , are then V_CsI_, , and , where the neutral state indeed turns out to be the most stable charge state under intrinsic conditions. Optimized structures of these defects are shown in Figure 1(a–c).

Following the same reasoning, we find the neutral compound interstitial defects [CsI]_i_ and , shown in Figure 1(d,e) through recombination of the point interstitials , , and . For larger potential compound interstitials, such as , we found that the lattice becomes too distorted and the DFE becomes very large.

**Figure 2:**
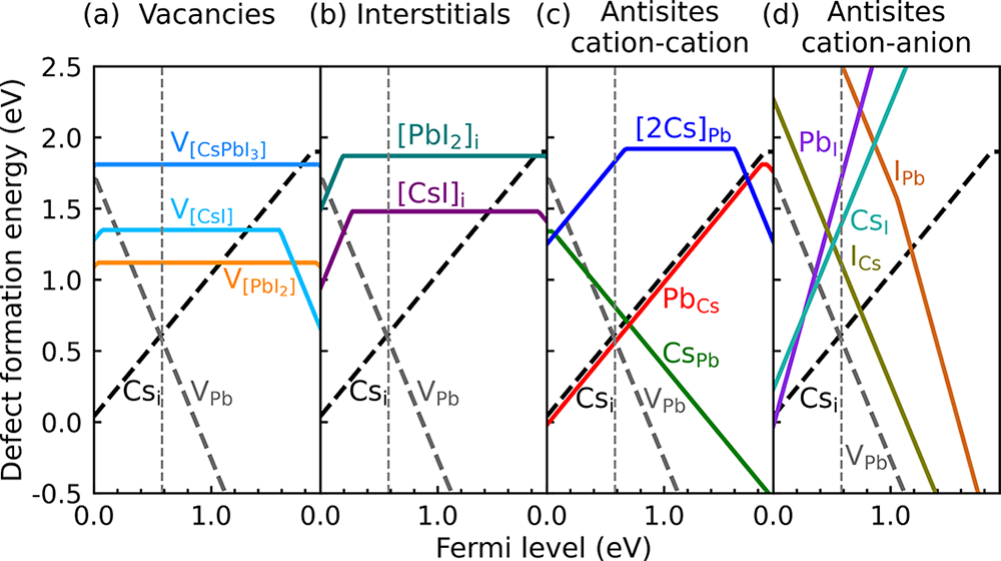
Formation energies of compound defects as a function of the Fermi level: (a) vacancies, (b) interstitials, (c) cation–cation antisites, and (d) cation–anion antisites. For comparison, the dashed black lines represent the formation energies of the two dominant point defects in CsPbI_3_. The intrinsic Fermi level ( eV), determined by the charge neutrality condition, eq 2, is indicated by the vertical dashed gray line.

Formation energies of the compound vacancies and interstitials, calculated according to eq 3, are shown in Figure 2(a,b). Taking into account of all point defects and compound defects, the intrinsic Fermi level, , calculated with the charge neutrality condition, eq 2, is 0.58 eV with respect to the VBM. At this condition  and  are the dominant atomic point defects,^22^ and the antisite , to be discussed below, is the most dominant compound defect. The compound vacancy and interstitial defects listed above, are then all stable in the neutral state. A list of the DFEs and concentrations, calculated at the intrinsic Fermi level, of these compound defects is given in Table 1.

**Table 1:** Formation Energies (Δ*H*_*f*_) and Concentrations of Compound Defects under Equilibrium Conditions (*c*_equilibrium_, *T* = 300 K); Recombination Reactions and Reaction Energies (Δ*H*_*r*_); and Concentrations of Compound Defects under Nonequilibrium Conditions (*c*_nonequilibrium_), All at the Intrinsic Fermi Level^a^

defects	Δ*H*_*f*_ (eV)	*c*_equilibrium_ (cm^–3^)	reaction	Δ*H*_*r*_ (eV)	*c*_nonequilibrium_ (cm^–3^)
Vacancies					
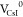	1.35	2.81 × 10^–1^	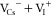	–0.06	7.75 × 10^8^
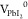	1.12	7.23 × 10^2^	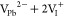	–0.83	5.26 × 10^14^
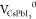	1.81	1.73 × 10^–9^	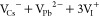	–1.54	5.10 × 10^13^
Interstitials					
[CsI]_i_^0^	1.48	1.51 × 10^–3^	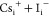	0.16	1.41 × 10^6^
[PbI_2_]_i_^0^	1.87	1.09 × 10^–9^	Pb_i_^2+^ + 2I_i_^–^	–0.30	3.24 × 10^5^
Antisites (Cation–Cation)					
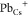	0.56	1.71 × 10^12^	Pb_i_^2+^ + V_Cs_^–^	–0.93	9.44 × 10^15^
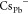	0.81	1.09 × 10^8^	Cs_i_^+^ + V_Pb_^2–^	–0.39	3.88 × 10^15^
[2Cs]_Pb_^+^	1.83	7.72 × 10^–10^	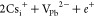	0.01	1.51 × 10^3^
Antisites (Cation–Anion)					
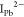	2.51	2.90 × 10^–21^	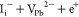	1.24	1.12 × 10^–12^
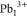	1.72	1.58 × 10^–7^	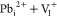	0.25	9.82 × 10^2^
I_Cs_^2–^	1.10	3.50 × 10^3^	I_i_^–^ + V_Cs_^–^	–0.31	1.10 × 10^13^
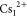	1.40	4.39 × 10^–2^	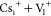	0.08	3.28 × 10^7^
Cation Pair Exchange					
Cs_Pb_Pb_Cs_^0^	1.02	2.74 × 10^4^	Cs_Pb_^–^ + Pb_Cs_^+^	–0.35	1.14 × 10^8^

a The specific nonequilibrium conditions are defined by defect formation at an elevated temperature equilibrium at *T* = 500 K, followed by allowing for recombination through isolation at *T* = 300 K.

A compound vacancy defect creates a considerable hole in the lattice, see Figure 1(a-c), and its DFE is correspondingly high. The vacancy  is relatively easiest to form, with a DFE of 1.12 eV, followed by V_CsI_ and , whose DFEs are 1.35 and 1.81 eV, respectively. All of these numbers are ≳0.5 eV higher than the DFEs of the simple point defects  and , which means that concentration of compound vacancy defects is negligible at room temperature under equilibrium conditions (Table 1).

Compound interstitial defects, [CsI]_i_ and , can be accommodated in the CsPbI_3_ lattice by a distortion or tilting of the Pb–I octahedra, see Figure 1(d,e), albeit at a considerable energy penalty, with DFEs of 1.48 and 1.87 eV, respectively. We conclude that compound interstitial defects also have negligible concentrations at room temperature under equilibrium conditions; see Table 1.

Turning to antisite defects, as there are two different cations in CsPbI_3_, antisites can be formed among cations, i.e., by a cation of one type occupying a position of a cation of the other type, Pb_Cs_ (Pb substitutes Cs) or Cs_Pb_; see Figure 1(f, g). We stick to the nomenclature of antisites, but note that replacing one cation by another can lead to a notable local distortion of the lattice, such that the substituting ion is significantly displaced from the lattice position of the original ion. Pb ions are nominally 2+, and Cs ions are 1+, so it is not surprising to find the most stable charge states of these antisites as  and . The DFE of  is comparable to that of the simple point defects  and , see Figure 2(c) and Table 1, which means that this antisite occurs relatively frequently under equilibrium conditions. The  antisite defect has a DFE that is ∼0.25 eV larger than that of , making it less favorable.

In principle it is possible that a Pb vacancy, , captures two Cs^+^ ions to form the [2Cs]_Pb_ antisite, see Figure 1(h). Somewhat surprisingly, the most stable charge state at the intrinsic Fermi level of this antisite is . Its DFE is, however, ≳ 1 eV larger than that of the  antisite, demonstrating that it is difficult to plant two Cs ions in one Pb lattice position; see Figure 1(g,h).

A second possible type of antisite results from placing an anion on a cation position, or vice versa. There are four possibilities, see Figure 1(i–l). Again we maintain the nomenclature of antisites, but note that the replacing anion or cation typically does not occupy a lattice site. For instance, in the I_Cs_ antisite the I ion does not replace the Cs ion at its lattice position (Figure 1(k)). Instead, it forms a Pb–I–Pb bridge bond nearby, which is a typical bonding configuration for I interstitials.^22^ In this sense, an antisite is actually a bonding configuration between a vacancy and an interstitial.

The most stable charge states of these antisites can be guessed from summing the charges of the point defects that can recombine to these antisites. For instance,  and  antisites originate from recombining , respectively  interstitials with  vacancies, whereas the  antisite results from recombining an  interstitial with a  vacancy.  is an exception to this rule; it might be a recombination between an  interstitial and a  vacancy. In general, cation–anion antisites lead to unusually high charge states for the defects inserted into the CsPbI_3_ lattice, Figure 1(i–l). This might in part explain their large DFEs, where all cation–anion antisite defects have a DFE that is at least 0.5 eV larger than that of simple point defects; Figure 2 and Table 1.

Finally, an exchange of two neighboring Cs and Pb cations leads to a defect that can be marked as Cs_Pb_Pb_Cs_. It can be thought of as formation of a compound defect between the cation–cation antisites  and . Consistent with that, the most stable charge state of Cs_Pb_Pb_Cs_ is the neutral state. Although in principle this compound defect is a simple exchange of a pair of Cs and Pb cations, its optimized structure involves a significant local distortion of the perovskite lattice, Figure 1(m). The  compound defect has a moderate DFE of 1.02 eV, Table 1, which is however significantly larger than that of the individual cation–cation antisites, implying that its concentration under equilibrium conditions is low.

In summary, under equilibrium conditions at room temperature, only the formation of cation(Cs)–cation(Pb) antisites is prominent, with  presenting a comparable concentration to those of the dominant point defects  and , and  is formed to a lesser extent. Other compound defects, antisites, compound vacancies or interstitials, are not favorable due to their large DFEs.

### Nonequilibrium Conditions

Defect concentrations can change drastically under nonequilibrium conditions. Highly nonequilibrium conditions typically occur during the growth of the perovskite crystals. The resulting concentration of defects can then not be simply deduced from the equilibrium relation (eq 1). The types and concentrations of defects that occur of course depend on the exact growth conditions. To estimate the potential role played by compound defects, we explore the following model.

It starts from the assumption that initially defects are created at an elevated temperature, which could reflect an annealing step during the growth process, for instance, with concentrations that can be estimated from eq 1. The crystal is then brought to room temperature, where the point defects and compound defects present are allowed to recombine or dissociate, according to eq 8, under the constraints of conservation of the total number of atoms in the defects, eq 10.

**Figure 3:**
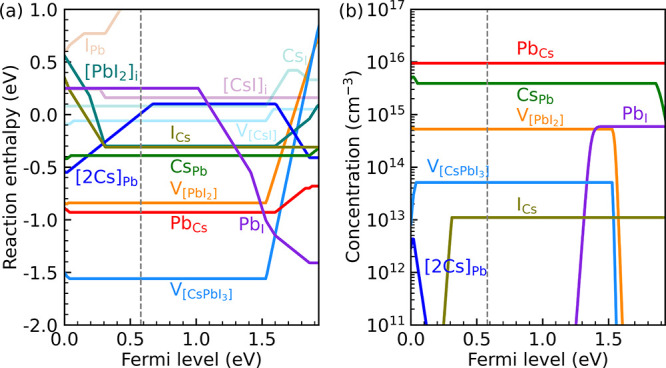
(a) Reaction energies of compound defects, eq 9, as a function of the Fermi level. (b) Concentrations under nonequilibrium conditions, resulting from the law of mass action at room temperature, eqs 8 and 10, with the initial concentrations of defects determined by equilibrium at *T* = 500 K. The intrinsic Fermi level ( eV) is indicated by the vertical dashed gray line.

A key parameter determining the recombination reaction is the reaction energy (eq 9). Table 1 shows the reaction energies, calculated at the intrinsic Fermi level, of the recombination reactions that lead to the compound defects, and Figure 3(a) shows the reaction energies as a function of the Fermi level. For a recombination reaction to lead to an appreciable concentration of a compound defect, its reaction energy needs to be significantly negative.

Figure 3(a) and Table 1 show that at the intrinsic Fermi level this is the case for the compound vacancies  and  and the antisite , with reaction energies in the range of −0.8 to −1.5 eV. The antisites  and , as well as the compound interstitial  and the cation pair exchange , have a moderately negative reaction energy between −0.3 and −0.4 eV, whereas that of the compound vacancy  is marginally small. The reaction energies of other compound defects, anion–cation antisites (except the mentioned ) and the double antisite , or the compound interstitial , are positive, which means that these complexes are not formed in significant concentrations.

However, merely having a negative reaction energy does not imply that a compound defect will form in an appreciable concentration, as formation of a complex necessarily involves a decrease in entropy. Using the law of mass action, eq 8, which is based upon free energies, the effects of entropy are included. At room temperature equilibrium conditions, the most prominent point defects are the Pb vacancy  and the Cs interstitial , with concentrations of 1.11 × 10^12^ and 5.03 × 10^11^ cm^–3^, respectively.^22^ Under those conditions, all compound defects have a concentration that is at least 3 orders of magnitude lower (see Figure S2 of the Supporting Information), which means that the loss of entropy involved in their formation reaction essentially prohibits the occurrence of compound defects. The antisite defect  is an exception, which forms in a large concentration of 1.71 × 10^12^ cm^–3^ resulting from its low formation energy rather than the recombination of point defects.

At *T* = 500 K the concentrations of the most prominent point defects,  and , and the cation–cation antisite  are raised to ∼10^16^ cm^–3^; see Figure S3 and Table S3 of the Supporting Information. Based on these initial conditions, the concentrations of compound defects at *T* = 300 K (and intrinsic Fermi level, eq 2), according to eqs 8 and 10, are shown in Figure 3(b). Most noticeable under these circumstances is that the two cation point defects recombine to form the antisite  in a large concentration of 3.88 × 10^15^ cm^–3^.

A third relatively prominent defect is the compound vacancy  with a concentration of 5.26 × 10^14^ cm^–3^. The compound vacancy  and the anion–cation antisite  occur at lower concentrations of 5.10 × 10^13^ and 1.10 × 10^13^ cm^–3^, respectively, whereas the concentrations of the other compound defects are much smaller (under intrinsic Fermi level conditions).

In summary, whereas at equilibrium conditions compound defects are unlikely to form at room temperature, creation of point defects at elevated temperatures and subsequent annealing leads to recombination of point defects, and a prominent appearance of cation–cation antisites  and . Less important, though still present in appreciable quantities, are the compound vacancies  and , and the anion–cation antisite .

### Shifting the Fermi Level

Nonequilibrium conditions of a different type occur when operating perovskite solar cells. Electrons and holes are produced by light absorption, creating quasi-Fermi levels for electrons and holes that are closer to the band edges than the intrinsic Fermi. The DFEs, eq 3, and therefore the defect concentrations, eq 1, are affected by the position of the Fermi level, depending on the charge states of the defects.

As can be observed in Figure 2(a,b), the compound vacancies and interstitials maintain their neutral states (and their DFEs) over a large range of Fermi level positions. Only if the Fermi level is close to the conduction band minimum (CBM) does V_CsI_ become negatively charged, and if the Fermi level is close to the valence band maximum (VBM), then [CsI]_i_ and  become positively charged.

The cation–cation antisite [2Cs]_Pb_, which is positively charged at the intrinsic Fermi level (Figure 2(c)), becomes neutral upon raising the Fermi level and becomes negatively charged for a Fermi level close to the CBM. The other cation–cation antisites behave similar to simple (charged) point defects, with  decreasing its DFE upon lowering the Fermi level, and  decreasing its DFE upon raising the Fermi level.

The DFEs of the highly charged cation–anion antisites of course depend strongly on the position of the Fermi level (Figure 2(d)). The  and  antisites become favorable for Fermi level positions close to the VBM, and the  and  become more important for Fermi levels close to the CBM.

At the intrinsic Fermi level, or indeed for a Fermi level positioned anywhere in the midgap region, we find that the most stable charge state of a compound defect is simply the sum of the charges of the point defects involved in the recombination reaction, eq 4:
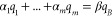
12As long as this holds, the reaction energy does not depend on the exact position of the Fermi level and is constant, see eqs 3 and 9, which can be observed in Figures 3(a) and S4. Consequently, for a Fermi level in this range, the concentrations of the compound defects do not depend upon the exact position of the Fermi level; see Figure 3(b).

If the Fermi level is close to the band edges, then defects change their charge states, as discussed above. In fact, charge conservation (eq 12) does not necessarily hold, as it becomes energetically more advantageous to accept holes or electrons from the valence or conduction bands by one or more of the defects involved in the reaction. Figure 3(a) shows that, as a result of this, reaction energies can change significantly if the Fermi level comes closer to the band edges. As an example, the reaction energy of [2Cs]_Pb_ decreases if the Fermi level either is close to the VBM or close to the CBM, where this compound defect becomes positively  or respectively negatively  charged.

Most remarkable in Figure 3(a) is the strong decrease of the reaction energy of the cation–anion antisite Pb_I_ if the Fermi level moves upward from 1.01 eV. At the intrinsic Fermi level, this compound defect is highly charged (, Figure 2(d)), but upon raising the Fermi level, it becomes energetically advantageous to capture one or more electrons from the conduction band and lower its reaction energy. Further noticeable is the strong increase of the reaction energies of the compound vacancies  and  for Fermi levels close to the CBM, and for the antisite I_Cs_ and compound interstitial  for a Fermi level close to the VBM. A detailed description of the reaction and defect formation energies of each compound defect is given in Figure S4 of the Supporting Information.

These changes of the reaction energies upon moving the Fermi level closer to the band edges, have consequences for the defect concentrations (Figure 3(b)). The cation–cation antisites Pb_Cs_ and Cs_Pb_ remain the dominant defect, but for a Fermi level close to the CBM (*E*_*F*_ > 1.6 eV), the concentration of the compound vacancies  and , which are third and fourth most important defects at midgap Fermi level positions, become negligible. The cation–anion antisite Pb_I_ becomes the third most important defect for *E*_*F*_ > 1.3 eV. For a Fermi level closer to the VBM not much happens, unless for the rather extreme case *E*_*F*_ < 0.1 eV, where the antisite [2Cs]_Pb_ begins to appear in non-negligible concentrations, while the anion–cation antisite I_Cs_ concentration becomes negligible.

### Charge State Transition Levels

**Figure 4:**
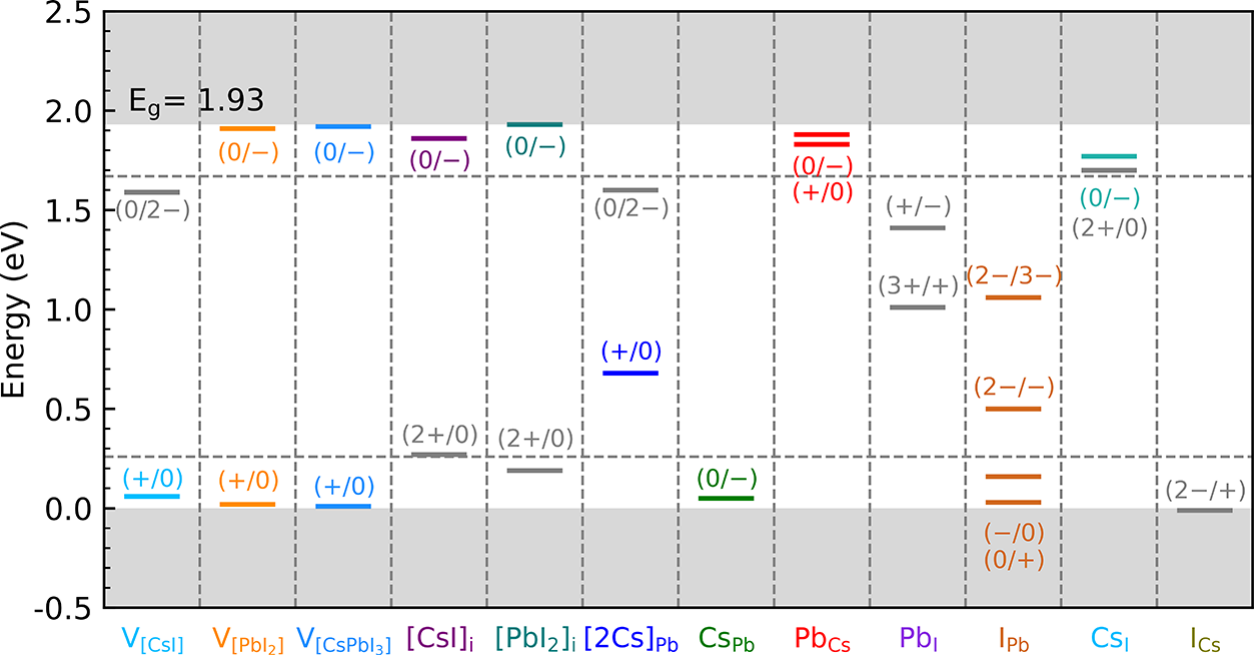
Charge state transition levels of compound defects in CsPbI_3_. The levels representing a change of a single ±e are indicated by colored lines. The bottom and top gray areas represent the valence and conduction bands (calculated with the SCAN+rVV10 functional without spin–orbit coupling). The two horizontal dashed lines are 10 *k*_B_*T* (*T* = 300 K) above the VBM and below the CBM, respectively.

Based on Figure 2 and eq 11, and accepting the caveats presented by DFT functionals, the CSTLs of compound defects are determined. The results for all defects considered in this paper, are shown in Figure 4. The most prominent compound defect, the cation–cation antisite Pb_Cs_, leads to double shallow donor levels, whereas the antisite Cs_Pb_ only leads to a shallow acceptor level. The compound vacancies  and  have both a shallow donor as well as a shallow acceptor level. The anion–cation antisite I_Cs_ has no levels inside the band gap.

At the intrinsic Fermi level, or indeed if the Fermi level is well inside the band gap, these are all compound defects that can occur in appreciable quantities; see Figure 3(b). If the Fermi level is close to the CBM, then the concentration of Pb_I_ antisites increases. Although this antisite introduces two deep levels inside the band gap, both of these levels involve a change in charge state of two electrons, i.e., 3+/1+ and +/–. These levels are likely to be much less active than donor or acceptor levels associated with a change of one in charge state, as the probability of trapping two electrons simultaneously is very low.^19,43^ If the Fermi level becomes extremely close to the VBM, then the concentration of [2Cs]_Pb_ becomes somewhat higher. As its CSTL (+/0) is well inside the band gap, this compound defect forms a deep trap, which can act as a recombination center.

Besides the defects discussed in the previous two paragraphs, all other defects occur in such negligible quantities, so that their electronic impact is negligible. In fact, the only compound defect considered in this paper that forms a series of deep trap levels, which is the anion–cation antisite I_Pb_ (Figure 3(b)), has a very large positive reaction energy (Table 1), so it does not form under practical conditions.

In summary, the relatively abundant compound defects either form shallow donor or acceptor levels (Pb_Cs_, Cs_Pb_, , , and I_Cs_) or electronically not very active levels (Pb_I_). Only under relatively extreme conditions, with a Fermi level very close to the VBM, the compound defect [2Cs]_Pb_ can form, which has a deep trap level.

## Conclusions

To conclude, we have studied the formation of compound defects in the archetype inorganic halide perovskite CsPbI_3_ by means of DFT calculations using the accurate and efficient SCAN+rVV10 functional. Considering compound vacancies, V_[CsI]_, , and , compound interstitials [CsI]_i_ and , cation–cation antisites Pb_Cs_, Cs_Pb_, and [2Cs]_Pb_, anion–cation antisites I_Cs_, I_Pb_, Cs_I_, and Pb_I_, and cation pair exchange Cs_Pb_Pb_Cs_, we evaluate their formation under equilibrium conditions and under conditions that reflect their formation as recombination reactions of simple point defects.

Although the energies of several of these recombination reactions are favorable, under equilibrium conditions at room temperature, only the formation of the antisite where Pb substitutes Cs is prominent, and the concentrations of point defects are too small to give any appreciable amount of other compound defects. However, under nonequilibrium conditions, mimicked by a high temperature annealing step, several types of compound defects can be formed in significant concentrations. Most prominent are the cation–cation antisites  and , with concentrations comparable to those of the dominant point defects  and . Smaller amounts of the compound vacancies  and  and the anion–cation antisite  can be observed, whereas the concentrations of other defects are negligible.

The formation energies and concentrations of compound defects in other halide perovskites will of course be different from those in CsPbI_3_. The same properties of point defects in hybrid organic–inorganic halide perovskites show clear trends upon changing the halide ions or the cations, and at least the Pb-based materials show qualitatively a similar behavior.^22^ It is therefore reasonable to assume that compound defects in these materials also show qualitatively similar properties to those in CsPbI_3_.

Under solar cell operating conditions the (quasi) Fermi level can shift to the proximity of the VBM and CBM, which promotes the formation of certain compound defects, and suppresses that of others. If the Fermi level is close to the CBM, then the formation of  and  is suppressed and that of the cation–anion antisite  is promoted, whereas if the Fermi level is close to the VBM, then the formation of  is suppressed and that of  is promoted. The other defects are less affected by a change in Fermi level.

The antisites and compound vacancies that can occur in appreciable concentrations (Pb_Cs_, Cs_Pb_, I_Cs_, , and ) tend to create shallow trap levels only. The antisite Pb_I_ creates several deep levels, which are, however, not very active electronically, as their charge state transition involves the arrival of two electrons simultaneously. The compound defect [2Cs]_Pb_ leads to a deep trap level. However, as discussed above, this defect is only slightly likely to form if the Fermi level is very close to the VBM. These results illustrate the exemplary electronic tolerance of halide perovskites toward the presence of defects.

## Supporting Information Available

List of convergence tests of the energy cutoff and size of supercell; number of possible defect sites and counting rule for each compound defect; concentration of compound defects at 300 K; temperature dependence of concentrations of point defects; table of concentrations of point defects at 300 and 500 K; detailed comparison of reaction energies and formation energies of each compound defect (PDF)
