# Supplementary material for: Compound Defects in Halide Perovskites: A First-Principles Study of CsPbI3
Source: J Phys Chem C Nanomater Interfaces. 2023 Jan 5;127(2):1189–97. doi: 10.1021/acs.jpcc.2c06789 (PMC9869396; doi:10.1021/acs.jpcc.2c06789)
Supplement: Supplementary file 1 — jp2c06789_si_001.pdf [file jp2c06789_si_001.pdf]

Supporting Information: Compound Defects in Halide Perovskites: A First-Principles Study of $CsPbI_3$

Haibo Xue,[†,‡] José Manuel Vicent-Luna,[†,‡] Shuxia Tao,[*,†,‡] and Geert Brocks[*,†,‡,¶]

†*Materials Simulation and Modelling, Department of Applied Physics, Eindhoven University of Technology, P.O. Box 513, 5600MB Eindhoven, the Netherlands.*

‡*Center for Computational Energy Research, Department of Applied Physics, Eindhoven University of Technology, P.O. Box 513, 5600MB Eindhoven, the Netherlands.*

¶*Computational Materials Science, Faculty of Science and Technology and MESA+ Institute for Nanotechnology, University of Twente, P.O. Box 217, 7500AE Enschede, the Netherlands.*

E-mail: s.x.tao@tue.nl; g.h.l.a.brocks@utwente.nl

Contents

1 Convergence tests S3

2 Density of possible defect sites S5

3 Concentrations of compound defects at 300 K S8

4 Temperature dependence of concentrations of point defects S9

5 Reaction energies and defect formation energies S10

References S11

1 Convergence tests

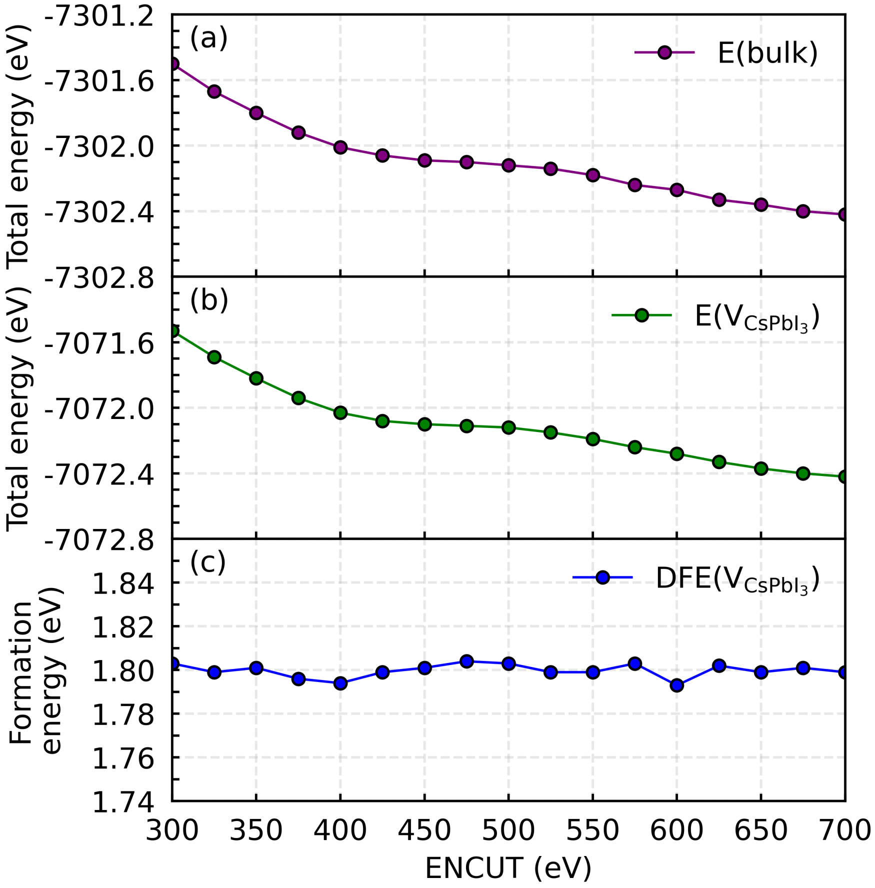

Figure S1: Convergence of the total energies and defect formation energies (DFEs) with respect to increasing the kinetic energy cutoff for the plane wave basis set. (a) and (b) Total energies of the pristine supercell and the defective supercell with the largest sized defect, vacancy $CsPbI_3$, respectively, (c) DFEs of the vacancy $CsPbI_3$

We have tested the convergence of DFEs with respect to increasing the kinetic energy cutoff for the plane wave basis set using the largest sized defect, vacancy $CsPbI_3$. Note that the values of the NGX, NGY, and NGZ parameters increase automatically upon increasing the cutoff energy. The results are shown in Figure S1. It demonstrates that the DFEs calculated with the cutoff used in the manuscript, 500 eV, are converged to within 0.01 eV.

Table S1: Convergence test of the defect formation energy with respect to the supercell size, using the largest sized defect vacancy $CsPbI_3$ as the example.

Supercell	Cell parameters $a \times b \times c$ (Å)	Cell Volume (Å^3)	ΔH_f ($V_{CsPbI_3}{}^0$) (eV)
$2 \times 2 \times 2$	$17.15 \times 17.73 \times 24.97$	7592	1.81
$3 \times 2 \times 2$	$25.73 \times 17.73 \times 24.97$	11391	1.84
$3 \times 3 \times 2$	$25.73 \times 26.60 \times 24.97$	17090	1.83

A convergence test of the defect formation energy with respect to the size of the supercell is conducted using the largest sized defect, vacancy $CsPbI_3$, during which the atomic positions of the defective supercell is relaxed. Starting from $2 \times 2 \times 2$, which is used for point defects in our previous work,[S1] the supercell size is expanded to $3 \times 2 \times 2$ and $3 \times 3 \times 2$, whereas the change in the formation energy of $V_{CsPbI_3}{}^0$ is subtle. Therefore, the $2 \times 2 \times 2$ supercell is considered to be sufficiently large for studying compound defects in this work, as it results in the best trade-off between accuracy and computational cost.

2 Density of possible defect sites

In the main text, $c_0(D^q)$ in Equations (6) and (7) defines the density of possible sites for the defect, including orientational degrees of freedom. It is calculated from

$$c_0(D^q) = \frac{n(D^q)}{V_{f.u.}}, \tag{S1}$$

where $n(D^q)$ is the number of possible sites and orientations for the defect D^q per formula unit of $CsPbI_3$, and $V_{f.u.}$ is the volume per formula unit, which is calculated to be 237 Å^3 or 2.37×10^{-22} cm^3. The values of $n(D^q)$ for each compound defect are given in Table S2.

Table S2: Number of possible sites for each type of compound defect and the counting rule used.

Defect	$n(D^q)$	Remarks
Vacancies		
V_{CsI}	3	1 Cs in the center of of the $CsPbI_3$ cube, with 12 possibilities for iodine anions in the middle of each edge to be an iodine vacancy, and each edge is shared by 4 cubes; so $1 \times 12/4 = 3$.
V_{PbI_2}	1	8 Pb cations at the corners of the cube, with each corner shared by 8 cubes; 2 iodine ions with one at the a-site and another at the e-site, and each of the two possibilities of combination is shared by two faces; so $8/8 \times 2/2 = 1$.
V_{CsPbI_3}	1	8 Pb cations at the corners of a cube, with three iodine ions forming a corner of a cube; so $8/8 \times 1 = 1$.
Interstitials		

Continued on next page

Table S2: Number of possible sites for each type of compound defect and the counting rule used. (Continued)

Defect	$n(D^q)$	Remarks
$[CsI]_i$	3	The Cs interstitial occupies a face of a $CsPbI_3$ cube, with 6 faces per cube, and each face shared by 2 cubes; the Cs interstitial is accompanied by an iodine interstitial occupies a site next to a lattice iodine anion in the middle of one of the four edges of a face, and each edge is shared by 4 cube; so $6/2 \times 4/4 = 3$.
$[PbI_2]_i$	6	The Pb interstitial occupies a face of a $CsPbI_3$ cube, with 6 faces per cube, and each face shared by 2 cubes; the Pb interstitial is accompanied by two iodine interstitials, with the I-Pb-I plane parallel to the in-plane or out-of-plane; so $6/2 \times 2 = 6$.
Antisites (cation-cation)		
Pb_{Cs}	1	1 Pb cation replaces the Cs cation in the center of a cube.
Cs_{Pb}	1	1 Cs cation replaces one of the 8 Pb cations at the corners of the cube, with each corner shared by 8 cubes; so $8/8 = 1$.
$[2Cs]_{Pb}$	1	Similar to the Cs_{Pb}.
Antisites (cation-anion)		
I_{Pb}	1	Similar to the Cs_{Pb}.
Pb_I	3	the Pb interstitial has a square pyramidal bonding to 5 surrounding I anions; the iodine anion in the middle of the edge opposite to the one where the iodine vacancy is serves as an apex of a pyramid, and all pyramids are inside the cube; so $12/4 = 3$.

Continued on next page

Table S2: Number of possible sites for each type of compound defect and the counting rule used. (Continued)

Defect	$n(D^q)$	Remarks
I_{Cs}	3	A Cs vacancy, with an iodine interstitial occupies a site next to a lattice iodine anion in the middle of each edge, and each edge is shared by 4 cube; so $1 \times 12/4 = 3$.
Cs_I	3	The Cs interstitial occupies a face of a $CsPbI_3$ cube, with 6 faces per cube, and each face shared by 2 cubes; the iodine vacancy can be one of the four lattice iodine ions in the middle of each edge surrounding the Cs interstitial, with each edge shared by 4 cubes; so $6/2 \times 4/4 = 3$.

3 Concentrations of compound defects at 300 K

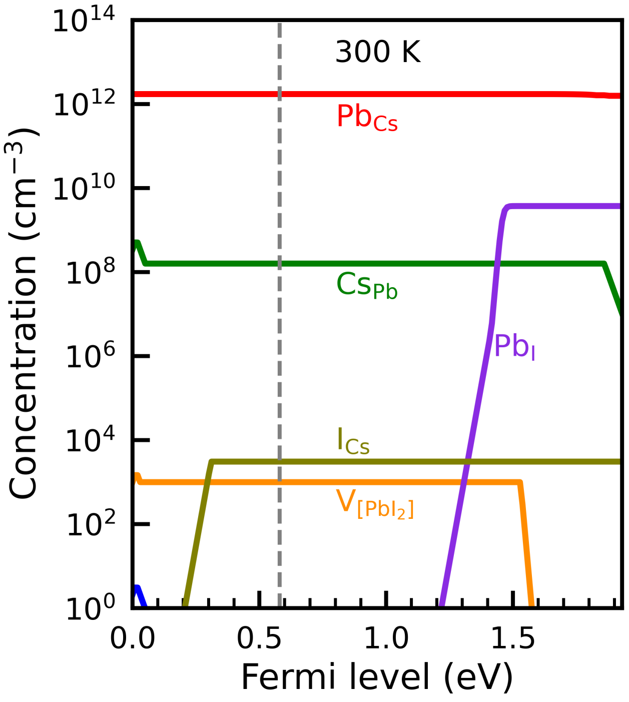

Figure S2: Concentrations resulting from the law of mass action at room temperature, with the initial concentrations of defects determined at equilibrium conditions at $T = 300$ K.

4 Temperature dependence of concentrations of point defects

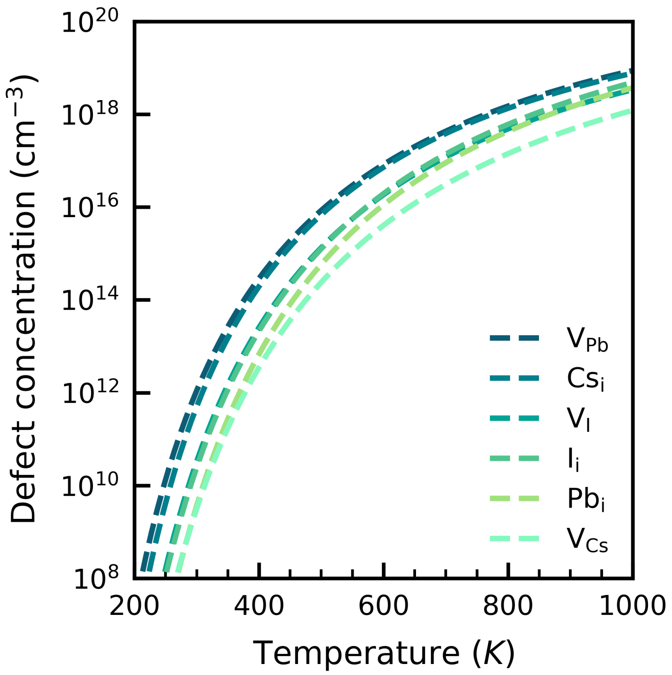

Figure S3: Equilibrium concentrations of point defects as a function of temperature.

Table S3: Equilibrium concentration of point defects calculated at 300 K and 500 K.

Defect	Concentration (cm^{-3})	
	300 K	500 K
$V_{Cs}{}^{-}$	3.21×10^{9}	2.40×10^{14}
$V_{Pb}{}^{2-}$	1.11×10^{12}	8.53×10^{15}
$V_{I}{}^{+}$	3.31×10^{10}	1.34×10^{15}
$Cs_i{}^{+}$	5.03×10^{11}	6.85×10^{15}
$Pb_i{}^{2+}$	3.70×10^{9}	5.89×10^{14}
$I_i{}^{-}$	2.48×10^{10}	1.27×10^{15}

5 Reaction energies and defect formation energies

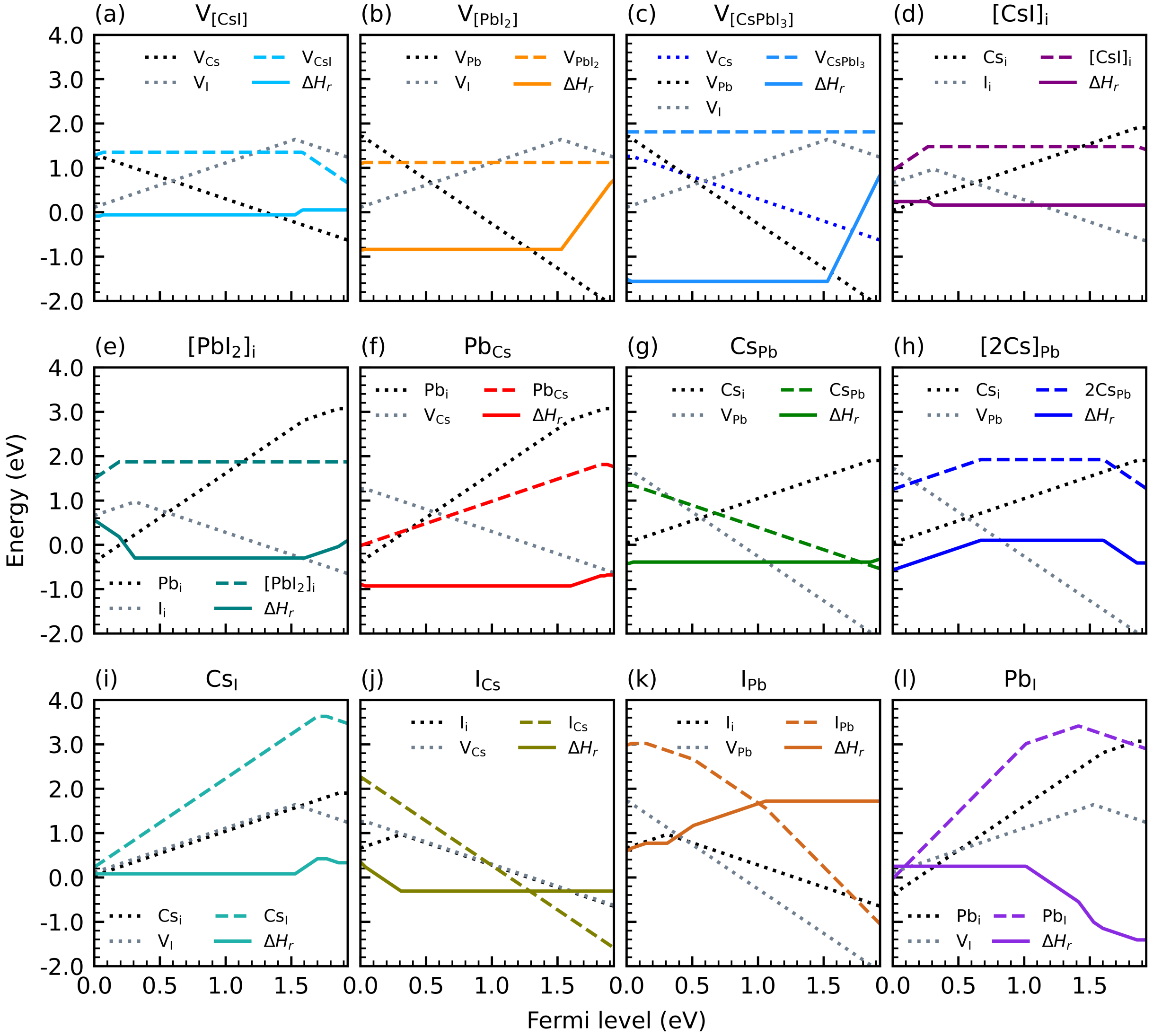

Figure S4: Reaction energies (ΔH_r) and defect formation energies (DFEs) as a function of Fermi level of compound defects. DFEs of the point defects are represented by dotted lines, while those of the compound defects are represented by dashed lines. The reaction energies are represented by solid lines.

References

(S1) Xue, H.; Brocks, G.; Tao, S. Intrinsic defects in primary halide perovskites: A first-principles study of the thermodynamic trends. *Phys. Rev. Mater.* **2022**, *6*, 055402.
